# Great Debate: Hyperthermic Intraperitoneal Chemotherapy for Colorectal Peritoneal Metastases—Should It Be Offered?

**DOI:** 10.1245/s10434-025-17651-9

**Published:** 2025-07-01

**Authors:** Jason M. Foster, Garrett M. Nash, Mecker G. Möller

**Affiliations:** 1 Division of Surgical Oncology, Department of Surgery, University of Nebraska Medical Center, Omaha, NE USA; 2 Colorectal Surgery Service, Department of Surgery, Memorial Sloan Kettering Cancer Center, New York, NY USA; 3 Department of Surgery, Section of Surgical Oncology, University of Chicago Pritzker School of Medicine, Chicago, IL USA

Colorectal cancer (CRC) stands as one of the most common malignancies worldwide, accounting for approximately 10% of all cancer cases and ranking as the third most prevalent cancer globally.^1^ Between 10 and 20% of patients develop colorectal peritoneal metastasis (CRC-PM) during their clinical course),^2,3^ causing a significant impact on the quality of life of these patients and making the disease more challenging to treat as it progresses. CRC-PM was historically considered a terminal condition.^4,5^ Over the past few decades, management approaches for CRC-PM have evolved considerably, particularly with the introduction of cytoreductive surgery (CRS) combined with hyperthermic intraperitoneal chemotherapy (HIPEC).^6,7^

The journey to recognizing CRS-HIPEC’s potential began with a 1992 observational study that reported a striking contrast: 20% survival rate at 2 years in the CRS-HIPEC group versus 0% in patients treated with chemotherapy alone.^8,9^ This finding, coupled with encouraging concomitant publication of positive outcome data on CRS-HIPEC application in appendix cancer, catalyzed global efforts to clarify the role of CRS with or without HIPEC for CRC-PM. In the following two decades, multiple retrospective studies reported better survival outcomes for patients treated with CRS-HIPEC—median survival ranging from 30 to 41 months compared with 15 to 25 months for CRS alone.^10–13^ Most HIPEC protocols employed either mitomycin C for 90–120 min or oxaliplatin for 30–90 min.

The field witnessed a watershed moment with the landmark Netherlands randomized trial, which compared CRS-HIPEC using mitomycin C for 90 min combined with best medical therapy (5-fluorouracil) against best medical therapy alone with palliative surgery. Results showed significantly improved median survival—22 versus 13 months (*p* = 0.03)—independent of the Peritoneal Cancer Index (PCI) score and cytoreductive surgery success.^14,15^ While this trial provided the first prospective evidence supporting CRS-HIPEC, it had notable limitations warranting further investigation, including the use of now-outdated chemotherapy regimens and uncertainty about HIPEC’s specific contribution to overall survival benefit over CRS alone.

## Gaps in Knowledge

As the field progressed, several high-volume centers published institutional data demonstrating impressive outcomes—CRS-HIPEC combined with contemporary chemotherapy achieved overall survival of 45–60 months for patients with optimal CRS.^16–19^ However, the landscape became more complex with the Prodige 7 trial, which specifically investigated oxaliplatin HIPEC efficacy by comparing CRS alone with CRS-HIPEC in patients receiving modern chemotherapy.^20^ The trial found no differences in overall survival (OS) or progression-free survival (PFS), suggesting that 30-min oxaliplatin HIPEC offered no additional benefit.

Many experts caution against broad extrapolation of these results to other HIPEC regimens, arguing that they do not justify abandoning mitomycin HIPEC administered for 60–120 min.^22^ Critics have identified key limitations in the Prodige 7 approach, including the use of potentially ineffective oxaliplatin and a HIPEC duration below the hyperthermic cytotoxic threshold necessary for therapeutic response.^8,23^ These insights, combined with preclinical studies and retrospective clinical evidence, highlight the urgent need for further trials to validate mitomycin-based HIPEC efficacy and explore additional metrics of HIPEC effectiveness.

The ongoing debate addresses several crucial questions regarding CRC-PM management, particularly concerning to the optimal applications of HIPEC in clinical practice.

## Primary Objectives of the Debate

With this important debate we aimed to:

1. Critically examine the current state of evidence regarding the efficacy, safety, and clinical impact of HIPEC in CRC-PM treatment.
2. Analyze gaps and inconsistencies in the existing data and identify areas that require further investigation to build a cohesive understanding of HIPEC’s role in CRC-PM treatment.
3. Propose pathways for future research, including the design of rigorous clinical trials, to study HIPEC efficacy and optimize patient outcomes.
4. Offer insight and recommendations for clinical practice and enhance the personalization of CRC-PM treatment strategies.

### PRO—Current Data Support Continued Use of HIPEC

#### State of the Evidence

In 1992 the first observational study evaluating the potential benefit of cytoreductive surgery (CRS) and hyperthermic intraperitoneal chemotherapy (HIPEC) in colorectal peritoneal metastasis (CRC-PM) reported a survival outcome benefit at 2 years: 20% in the CRS-HIPEC versus 0% (no survivors) for patients treated with chemotherapy, *p* = 0.05.^8^ This study, along with increasing publication of promising data of CRS-HIPEC improving survival outcomes in appendix cancer, catalyzed a global effort to further define the role of CRC ± HIPEC for CRC-PM. Over the next two decades following publication of this sentinel study, which identified two import prognostic factors in CRS-HIPEC outcome—optimal cytoreduction (CC0/CC1, R0-R2a) and disease burden (PCI)—several retrospective studies suggested improved outcomes for patients treated with CRS ± HIPEC compared with historical controls.^10^ The majority of the data published were for treatment with CRS-HIPEC but there were smaller studies reporting data with CRS alone. Notably, while not directly comparable, the published data for CRS-HIPEC consistently reported higher median survival 30–41 months compared with 15–25 months with CRS alone in series with similar systemic chemotherapy and rates of optimal cytoreduction (Table 1).^10–12^ While the list of potential HIPEC agents is extensive (Table 2), the majority of the retrospective HIPEC studies used HIPEC with mitomycin for 90–120 min or oxaliplatin for 30–90 min.

**Table 1:** Outcome of CRS alone & CRS-HIPEC Retrospective studies 2004–2014

	CRS ALONE					CRS-HIPEC					
Author Year	# Pts	Sys. Tx	PCI	CC 0/1 (%)	OS (mo.)	OS (mo.)	Author Year	# Pts	Sys. Tx	PCI	CC 0/1( %)
Elias 2004	19	5-FU	15-25	100	25	32	Glehen 2004	377	>60%	NR	100%
Scaringi 2009	27	NR	NR	82%	15	33	da Silva 2006	70	NR	14	100%
Mulsow 2011	31	5-FU, oxal/iri	NR	100	22	30	Elias 2010	523	50%	NR	84%
Kobayashi 2010	20	5-FU	NR	100	24	37	Ung 2013	109	NR	NR	100%
Kobayashi 2014	484	5-FU, oxal/iri	NR	100	**25**	**41**	Esquivel 2014	705	NR	NR	82%

**Table 2:** Hyperthermic intraperitoneal chemotherapy (HIPEC) agents

Mitomycin
Oxaliplatin
Melphalan
Cisplatin
Carboplatin
Paclitaxel
Docetaxel
Doxil
Doxorubicin
Etoposide
Floxuridine (FUDR)
Etoposide
Gemcitabine
Drug combinations

The first randomized trial conducted in the Netherlands compared CRS-HIPEC using 90 min with mitomycin HIPEC combined with best medical therapy (5-fluorouracil) versus best medical therapy alone with selective use of palliative surgery. It was a positive trial reporting improved median survival of 22 versus 13 months (*p* = 0.03) for patients receiving CRS-HIPEC and was independent of PCI score and optimal CRS.^14^ The subset analysis revealed that patients who achieved CCO (no visible disease) or CC1 (residual disease < 2.5 mm) experienced the largest survival benefit of 48 months and 20 months, respectively.^15^ This landmark trial provided the first prospective evidence of CRS-HIPEC benefit. Specifically, the combination of optimal surgery (CC0/1) combined with 90 min of mitomycin HIPEC was an effective surgical intervention that improved overall survival (OS) compared with medical therapy alone. However, there were two major limitations of the trial design that required further exploration. The first was use of 5-FU chemotherapy as the development of better systemic therapy than 5-FU alone (FOLFOX, FOLFIRI, with and without biologic) emerged during the course of the trial. The second was whether the CRS alone was driving survival benefit since it was not clear from this trial how much HIPEC contributed to survival, particularly in the setting of better systemic therapy. Following the publication of the Netherlands trial, several high-volume centers published institutional outcome data demonstrating that CRS-HIPEC in conjunction with contemporary chemotherapy achieved OS of 45–60 months for patients who achieved optimal CRS.^12,16,24^ A French case-controlled study that compared 48 prospectively managed patients with CRC-PM treated with contemporary chemotherapy and CRS-HIPEC against 48 matched retrospective patients with CRC-PM treated with contemporary chemotherapy alone, demonstrated median survival 60 versus 24 months, *p* = 0.05, respectively.^18^

The second randomized HIPEC trial completed, Prodige 7, was designed to directly determine the efficacy of oxaliplatin HIPEC for 30 min by comparing CRS alone with CRS-HIPEC in patients treated with best contemporary chemotherapy.^20^ The trial reported no difference in OS (41 months for both groups) and PFS (11 versus 13 months), thus establishing that HIPEC with oxaliplatin 30 min did not have efficacy in CRC-PM. These results were not unexpected and were predicted by preclinical studies that reported efficacy of 90-min mitomycin HIPEC in organoids, while no efficacy was observed with 30-min oxaliplatin HIPEC, which also was supported by retrospective clinical data demonstrating oxaliplatin yielded worse OS compared with mitomycin.^22,23,25^ Specifically, Spiliotis et al. evaluated outcomes in matched patients on the basis of median PCI (15 versus 16), rates of neoadjuvant chemotherapy (63 versus 78%), and optimal CC0/1 (83% versus 79%) and reported that OS was 54 versus 26 months, *p* = 0.01, for mitomycin versus oxaliplatin HIPEC, respectively.^25^ While the results of Prodige do not support the further use of oxaliplatin HIPEC, these results cannot be extrapolated to other HIPEC regimens and do not provide evidence to discontinue mitomycin HIPEC for 60–120 min.^13,20,21^ Finally, it is important to briefly highlight HIPEC data in the prophylactic setting for high-risk CRC. Two trials studied oxaliplatin 30-min HIPEC (COLOPEC and PROPHYLOCHIP) and both similarly failed to demonstrate efficacy measure by a reduction peritoneal metastasis; HIPECT4, which utilized mitomycin HIPEC, reported improved peritoneal control (HR 0.21, *p* = 0.03), providing the first prospective clinical data mitomycin HIPEC efficacy in CRC.^26–28^

### Present Considerations

The Prodige 7 trial should be acknowledged as a pivotal trial in the field by providing accurate prospective data regarding OS and PFS for the surgical management of CRC-PM with contemporary chemotherapy. Importantly, the reported median survival of 41 months significantly exceeds any reported survival for CRC-PM achieved with chemotherapy alone. The flaws of the study include utilizing 30-min oxaliplatin HIPEC, a regimen that lacks efficacy,^22,23,25^ and a HIPEC duration below the threshold of the hyperthermic cytotoxic window for triggering a therapeutic response (60–120 min). Both have been highlighted by critics of the trial as well as the Prodige study team.^13,20,21^ A recent survey by PSOGI demonstrated that the Prodige trial has shifted most providers to mitomycin HIPEC for 90–120 min.^16^ The utilization of mitomycin HIPEC is supported by both preclinical and robust level 2 data and it warrants the development of clinical trials to validate, define, and quantify efficacy in the management of CRC-PM. There has been sufficient evidence regarding improved OS with mitomycin compared with oxaliplatin to support a trial to similar to Prodige with OS as the primary endpoint.

**Fig. 1:**
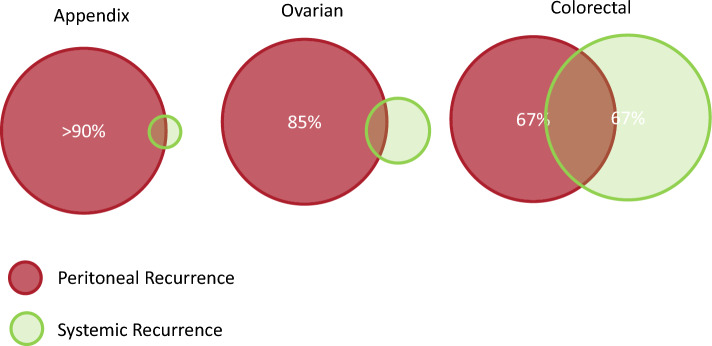
Appendix and ovarian have a low risk of extraperitoneal recurrence while colorectal has an equal risk of peritoneal and extraperitoneal recurrence.

However, in the development of trials to test the efficacy of mitomycin HIPEC, it is important to recognize that extraperitoneal patterns of recurrence in patients with CRC-PM will directly impact OS outcomes, but it is not clear how much of this impacts disease-specific survival. Therefore, exploration of other metrics of HIPEC efficacy that directly impact patients with CRC-PM is equally important in clinical trials to fully understand how HIPEC may benefit patients with CRC-PM independent of impact on OS. Unlike other prototypical peritoneal surface malignancies (appendix, ovarian) where more than 85–90% of first recurrences are peritoneal and are the primary driver of disease-specific survival, patients with CRC-PM have an equal chance of peritoneal versus extra-peritoneal first recurrence, which will influence disease-specific overall survival (Fig. 1).^16,24^ Since extraperitoneal events are not mitigated by HIPEC, it is conceivable that HIPEC is able to yield direct clinical benefits in CRC-PM, independent of demonstrable overall survival benefit. Hassan et al. recently provided evidence of the competing challenges of extraperitoneal recurrence in 146 patients with CRC-PM. This study reported that while DFS survival was 12 months, the peritoneal progression-free survival (PerPFS) was 25 months.^29^ While the reported DFS aligned with Prodige’s 11–13 months, the PerPFS was longer at 25 versus 15–17 months, with more than 70% of 146 patients receiving HIPEC with mitomycin. Delhorme et al. specifically compared PerPFS oxaliplatin (*n* = 52) versus mitomycin (*n* = 85) HIPEC and reported superior PerPFS with mitomycin HR 0.59 (*p* = 0.008).^30^ Both studies provide compelling evidence that PerPFS is an essential secondary endpoint to be included in any future CRC-PM trial.

While PerPFS will capture the time to peritoneal recurrence, the amount of tumor burden at recurrence is an equally important metric that needs to be captured and fully characterized to determine HIPEC efficacy. While radiographic peritoneal disease burden is important, currently there is no standardized scoring system, nor has radiographic recurrence burden been linked directly to patient outcome. However, there are some simple but important clinical endpoints that can be included and collected as secondary endpoints in clinical trials, including the rates of symptomatic disease due to ascites, bowel obstruction, and pain due to peritoneal recurrence. One other endpoint that reflects recurrent tumor burden is the ability and frequency to perform an iterative CRS (iCRS) upon peritoneal recurrence, which more recently has been determined to be a major factor in the long-term survival of patients with CRC-PM.^19^ One of the largest studies consisting of 414 patients with CRC-PM who underwent optimal cytoreduction at index reported that 25% had peritoneal recurrence only and 41% of these patients underwent a subsequent optimal iCRS. The survival for optimal iCRS patients was 27 versus 8 months for the 59% of patients not amenable to optimal iCRS (*p* = 0.001).^31^ These data, along with other series, highlight the frequency of iCRS in the management of CRC-PM and how it can potentially be another surrogate endpoint for peritoneal tumor burden in future trials.^19,31,32^ Additionally, they demonstrate how iCRS contributes to individual CRC-PM long-term survival and may be another metric that reflects tumor burden.

### Future

There are currently two active clinical trials investigating the efficacy of mitomycin HIPEC. CAIRO 6 is the third trial to open and recently reported phase II data demonstrating the safety of neoadjuvant chemotherapy in the Netherlands, where currently CRS-HIPEC alone is considered the standard of care for resectable CRC-PM.^33,34^ In this progressive phase III trial, CRS-HIPEC with 90-min mitomycin alone is the control arm and the experimental arm is neoadjuvant chemotherapy, CRS-HIPEC, and adjuvant chemotherapy. While this provocative trial will provide the first landmark prospective data for the outcomes of patients managed exclusively with surgery at diagnosis, it will not provide answers to the central question, which dates back to the initial Netherlands trial, regarding whether HIPEC provides clinical benefit for patients managed with contemporary chemotherapy and optimal CRS.

The GEOCOP trial will investigate efficacy of 60 min of mitomycin HIPEC by recording relapse-free survival in patients who receive best systemic therapy and CRS with no residual disease (CCO).^35^ Specifically, it is powered to detect 3-year RFS of 70% for CRS-HIPEC versus 50% for CRS alone. While this trial will provide direct evidence of mitomycin’s efficacy, it is not powered to provide evidence of the impact on OS, which has been a focal point for critics of this therapy. While proving direct efficacy is a central scientific question, it will also be important to demonstrate that this improvement has clinical impact for patients on the basis of tumor burden and quality of life.

There is well-established preclinical and clinical evidence supporting the efficacy of 90–120 min of mitomycin HIPEC, which requires validation of this efficacy in randomized clinical trials. While the ongoing trials will provide important data, additional well-designed trials based on OS and enriched with secondary endpoints (PFS, PerPFS, symptomatic PR, iCRS) will ultimately be required to convince critics. Until these trials have been completed, the current data supports the continued use of HIPEC other than 30-min oxaliplatin.

Finally, it is imperative to recognize that the heterogeneity of CRC-PM impacts both the response to systemic chemotherapy and HIPEC agents. This will challenge efforts to define HIPEC efficacy in clinical trials and can lead to the identification of CRC-PM subsets that benefit some more than others. The future development of organoid, xenograft, and orthoptic models that can be integrated into clinical trials, along with development of surrogate predictive tools, such as mutation and gene profiles as well as circulating cells, DNA, and/or exosomes, will be powerful tools in the future to help establish personalized care models.

### CON—Review of Conflicting Data of Great Value

According to the 2023 National Comprehensive Cancer Network guidelines, “complete cytoreductive surgery (CRS) and/or intraperitoneal chemotherapy (IPC) can be considered in experienced centers for selected patients with limited peritoneal metastases for whom R0 resection can be achieved. However, the significant morbidity and mortality associated with [hyperthermic intraperitoneal chemotherapy] HIPEC, as well as the conflicting data on clinical efficacy, make this approach very controversial.” These “conflicting data” on the use of HIPEC for peritoneal metastasis of colorectal origin (pmCRC) make this debate unlikely to resolve basic disagreements between the contrasting perspectives. Nevertheless, this exercise is of great value so that clinicians can place the recently reported trials in context.

Prior to any prospective trials attempting to establish the efficacy of regional therapy for pmCRC, many clinicians interpreted that there was a clinical benefit of CRS/IPC on the basis of retrospective data and prospective single-arm studies. However, correlation is not causation, and it is not appropriate to use such data as “proof of efficacy” or “clinical benefit.” Many examples of inappropriate interpretation of uncontrolled data exist. One dramatic example is the use of hormone replacement therapy (HRT), which was prescribed to 90 million U.S. women in 1999.^36^ Initially, HRT was promoted as a method of addressing the symptoms of menopause. However, the increased use of HRT was partially based on non-randomized studies that suggested that HRT would reduce the risk of a cardiovascular disease event. However, the Women’s Health Initiative (WHI) randomized trial subsequently demonstrated that “…health risks [of HRT] exceeded benefits…” with respect to coronary artery disease, stroke, and pulmonary embolism.^37^ As a result, the use of HRT dramatically dropped and it is widely accepted as ineffective with respect to reducing risk of morbidity and mortality of a variety of cardiovascular endpoints. When considering the existing data for and against HIPEC, one must keep this sobering lesson in mind.

The first prospective data supporting the use of regional therapy with CRS and HIPEC were the Dutch Trial, published in 2003 before the advent of combination systemic chemotherapy.^38^ This well-known study of 105 patients with pmCRC reported that the group randomized to CRS-HIPEC (with mitomycin) and systemic 5FU had superior median survival to those randomized to 5FU alone (22.3 versus 12.6 months, *p* = 0.03). The investigators observed that survival benefit was dependent on the success of achieving an optimal CRS, as those who had a CC0 resection had a 48-month median survival compared with 20 months after CC1 resection and just 5 months after CC2/3 resection. It also demonstrated that this surgery can be high risk, with an 8% perioperative mortality and 14% enteric fistula rate in the experimental arm. The latter conclusions led to an understanding of the importance of patient selection for this surgery and value of centralization of care to surgically experienced centers to lower the risk of unacceptable morbidity. Similarly, the Swedish Trial demonstrated that CRS with postoperative intraperitoneal 5FU could produce better overall survival than the arm that received only FOLFOX (HR 0.51, 95% CI 0.27–0.96).^39^

These trials contributed to the widespread adoption of CRS/IPC, though controversy remains, as prior to 2021, there were no randomized trials that attempted to isolate the impact of intraperitoneal chemotherapy. Nevertheless, numerous retrospective series reported morbidity outcomes and survival rates that appeared better than historical controls. However, use of historical controls is complicated by the stepwise improvement in overall survival for patients treated in the current era, who can expect median survival greater than 2 years with sequential combination therapy compared with 1 year in the 5FU era.^40^

The most important trial in the current era is the randomized, phase III multicenter PRODIGE 7 trial, which reported results from 265 patients with pmCRC who received perioperative standard 5FU-based systemic chemotherapy and optimal CRS.^20^ They were randomized to HIPEC with oxaliplatin or no additional therapy. This study reported identical OS, (41.7 months in HIPEC arm versus 41.2 months in the non-HIPEC arm) and a 60-day grade 3–5 morbidity rate, which was significantly higher in the HIPEC arm (26% versus 16%; *p* = 0.035). The authors concluded that “cytoreductive surgery alone should be the cornerstone of therapeutic strategies with curative intent for colorectal peritoneal metastases.”

The other published study of note, PROPHYLOCHIP-PRODIGE 15, is a phase III trial of 150 patients with (1) synchronous, localized pmCRC removed during the primary tumor resection, (2) resected ovarian metastases, or (3) a perforated primary tumor.^41^ This trial demonstrated that the group randomized to second-look surgery plus oxaliplatin-HIPEC had similar 3-year DFS compared with patients who underwent surveillance only (44 versus 53%, *p* = 0.82). Not only did the HIPEC arm fail to demonstrate superior survival, but 41% of patients in that arm experienced high-grade complications at the time of their second look surgery as well, including 11% of that arm returning to the operation room for anastomotic or bleeding complications. The authors concluded that “second-look surgery plus oxaliplatin-HIPEC did not improve disease-free survival compared with standard surveillance.”

A 2023 American Society of Clinical Oncology (ASCO) presentation by this author reported a randomized clinical trial, ICARuS NCT 01815359, comparing recurrence-free survival (RFS) after HIPEC with mitomycin for 100 min or early postoperative intraperitoneal chemotherapy (EPIC) with floxuridine^42^ (Alexander J. Rossi, MD, 2021). This trial demonstrated no difference in 3-year RFS between the HIPEC and EPIC groups, (10 versus 23%, *p* = 0.14) [publication pending]. Though this study had no placebo arm, the outcomes in the HIPEC arm was indistinguishable from the HIPEC arm of Prodige 7, and suggest that the lack of efficacy of HIPEC in Prodige 7 is not related to an ineffective agent, oxaliplatin, or to inadequate duration of therapy, 30 min, and is consistent with a lack of efficacy of mitomycin when delivered into the intraperitoneal space. This is hardly surprising, as oxaliplatin is an active drug when given systemically for pmCRC and mitomycin is not effective systemically.

What can we conclude from these clinical trials? They appear to support the use of regional therapy with CRS; however, the addition of HIPEC has not demonstrated a survival benefit compared with other forms of intraperitoneal therapy or to use of CRS alone for pmCRC. One can cherry pick data from post hoc analyses and find groups that may have comparatively better outcomes. For example, the mid-range PCI cohort in Prodige 7 suggested a possible differential survival outcome of HIPEC. However, post hoc analyses can only be considered hypothesis generating for future studies and cannot guide standard of care recommendations, as they were not the primary objectives of the studies and subject to the multiple testing problem; the more inferences are made, the more likely erroneous inferences become, and correlation is not causation.

### Debate Summary

#### Efficacy and Safety of HIPEC

Proponent’s Position: Dr. Foster emphasizes retrospective data showing the better survival outcomes of HIPEC when combined with CRS over CRS alone, particularly with the use of mitomycin C. The Netherlands trial demonstrated a significant survival advantage with CRS-HIPEC, with some reports of median survival of up to 60 months in patients achieving optimal cytoreduction.^8,10,14,18^

Contrary’s Position: Dr. Nash highlights that retrospective studies can produce spurious results and that there are no prospective data that isolate benefit of HIPEC, compared with CRS alone He underscores the findings from the Prodige 7 trial, which showed no difference in overall survival and higher complications in the HIPEC arm, and no relative benefit of HIPEC with mitomycin C over other IPC regimens, thus questioning the added benefit of HIPEC in the contemporary chemotherapy setting.^20^

#### Limitations of Existing Studies

Proponent’s Position: Dr. Foster argues that the flaws in the Prodige 7 trial, such as the use of 30-min oxaliplatin HIPEC (a regimen that in preclinical and clinical retrospective studies has shown lower efficacy compared with mitomycin C^22,23,25^ and the shorter HIPEC duration, limit the generalizability of its results. Further, he advocates for renewed focus on optimal HIPEC protocols and agents better suited for efficacy.^21,22,29^

Contrary’s Position: Dr. Nash continues to emphasize the importance of evidence-based practice, drawing attention to historical examples such as hormone replacement therapy for cardiovascular disease, where early enthusiasm based on uncontrolled data was later disproven by larger randomized controlled trials.^36,43^

#### Future Directions and Clinical Trials

Proponent’s Position: Dr. Foster calls for robust, well-designed clinical trials that incorporate additional endpoints such as peritoneal progression-free survival (PerPFS) to fully capture HIPEC’s benefits beyond overall survival. He highlights ongoing trials such as CAIRO 6 and GEOCOP, which are expected to provide crucial insights into the benefits of mitomycin HIPEC.^34,35^

Contrary’s Position: Dr. Nash stresses that we have insufficient data to conclude that HIPEC offers benefit, and it remains an experiment therapy for CRC-PM. There is a need for harmonized methodologies, better patient selection criteria, and more comprehensive data on quality of life (QoL) and long-term outcomes to assess the net benefit and risks associated with HIPEC.^44,45^

#### Personalized Care and Heterogeneity of CRC-PM

Both sides recognize the heterogeneity of CRC-PM and the necessity for personalized treatment approaches. They advocate for the integration of predictive tools such as genetic profiles and circulating biomarkers into clinical trials to identify patient subsets that may benefit the most from HIPEC and tailor treatments accordingly.^22^

### Important “Take-Away” Messages

- *Efficacy of Mitomycin C*: Retrospective studies suggest efficacy of mitomycin-C-based HIPEC as part of CRS for CRC-PM; however, further prospective trials are needed.
- *Contextualizing Prodige 7 Findings*: While Prodige 7’s findings are significant; its limitations highlight the need for continued investigation into optimal additional therapies to use in conjunction with CRS protocols..
- *Comprehensive Metrics for Assessment*: Future trials should incorporate a range of metrics, including PerPFS, QoL, and symptomatic disease, to provide a holistic assessment of benefits and limitations of treatments in CRC-PM management.
- *Centralized Expertise and Patient Selection*: Centralizing surgical management of pmCRC in experienced centers and employing stringent patient selection criteria remain crucial to minimizing complications and optimizing outcomes.
- *Future Directions in Personalized Care*: The future of CRC-PM treatment lies in personalized care models, integrating advanced predictive tools and trial designs to tailor treatment strategies and improve patient outcomes.
